# Intra-antral application of an anti-fungal agent for recurrent maxillary fungal rhinosinusitis: a case report

**DOI:** 10.1186/1752-1947-6-245

**Published:** 2012-08-20

**Authors:** Adekunle D Dunmade, Olushola A Afolabi, Biodun S Alabi, Segun Segun-Busari, Olubisi A Koledoye

**Affiliations:** 1 Department of Otorhinolaryngology, University of Ilorin Teaching Hospital, Ilorin, Nigeria

**Keywords:** Fungal sinusitis, intra-antral application, recurrence, surgical debulking

## Abstract

**Introduction:**

Fungal infection of the paranasal sinuses is an increasingly recognized entity both in immunocompetent and immunocompromised individuals. Treatment has been via use of either surgical or medical modalities, or a combination of the two. Here, we present a case of utilization of intra-antral application of an anti-fungal agent in the management of recurrent fungal sinusitis in an indigent Nigerian patient.

**Case presentation:**

We present the case of a 30-year-old West African Yoruba man, an indigent Nigerian clergyman, who presented to our facility with a history of recurrent nasal discharge (about one year), recurrent nasal blockage (about five months), and right facial swelling (about one week). After intra-nasal antrostomy for debulking with a systemic anti-fungal agent, our patient had a recurrence after four months. Our patient subsequently had an intra-antral application of flumetasone and clioquinol (Locacorten®-Vioform®) weekly for six weeks with improvement of symptoms and no recurrence after six months of follow-up.

**Conclusions:**

We conclude that topical intra-antral application of anti-fungal agents is effective in patients with recurrent fungal maxillary sinusitis after surgical debulking.

## Introduction

Fungal rhinosinusitis is a form of chronic rhinosinusitis (CRS), which is an inflammatory disorder of the nose and paranasal sinuses. It is clinically defined as persistence of symptoms of nasal blockage or discharge for at least 12 weeks combined with endoscopic abnormalities (polyps, mucopurulent discharge, mucosal swelling) or an abnormal sinus on computed tomography (CT) scan findings; it may be primary or secondary fungal infection. Other symptoms may include facial pain or a reduced sense of smell [1]. While allergy and bacterial infection play a role in the etiology of this clinical condition, it is best considered as a multi-factorial chronic inflammatory disorder. It is distinguished from allergic rhinitis by the involvement of both the nose and the paranasal sinuses.

Fungal infection of the paranasal sinuses is an increasingly recognized entity both in immunocompetent and immunocompromised individuals. It is not an uncommon condition. In patients who are immunosuppressed, the fungus is typically invasive and can manifest in an acute invasive or a more chronic invasive form [2]. The treatment for this is well documented in the literature and consists of a combination of surgical and medical therapy, including systemic anti-fungal therapy. Invasive fungal disease is a unique entity and represents angio-invasive fungal propagation in the immunocompromised host setting. This is not the common mode of presentation of chronic rhinosinusitis of bacterial origin in the vast majority of patients with chronic sinus issues. In patients who are immunocompetent, fungus may be involved in several forms. Fungal ball development is well documented and was previously termed mycetoma. It is a slow-growing fungal collection in an abnormal sinus. Surgical evacuation is a definitive treatment for this condition.

Anti-fungal agents can be used as systemic medications or as topical preparations delivered directly to the sinuses. Systemic anti-fungals are given orally or intravenously. Topical treatments can be given using different delivery systems. Topical therapy may be administered by douching, nebulization, atomization, inhalations, irrigation, spray, drops or powder insufflations. There is a significant difference in the distribution of medication to the paranasal sinuses before and after sinus surgery [3]. Early diagnosis is vital to reduce morbidity and avoid vascular invasion and its associated complications. We evaluated the utilization of intra-antral topical anti-fungal agent application in the management of recurrent inflammatory fungal maxillary sinusitis in an indigent Nigerian patient.

## Case presentation

We present the case of a 30-year-old West African Yoruba man, an indigent Nigerian clergyman, who presented to our facility with recurrent nasal discharge of a history of about one year, recurrent nasal blockage of a history of about five months, and right facial swelling of a history of about one week. The nasal discharge was bilateral, mucopurulent, brownish, foul smelling, associated with excessive sneezing accompanied with intermittent bilateral nasal obstruction five months prior to presentation. The obstruction started from the right nasal cavity and subsequently involved the left side to become bilateral with an associated inability to perceive smell and no history of epistaxis. The right cheek swelling was noticed a week prior to presentation, progressive and associated with facial pain. A nasal examination revealed a pale polypoidal mass in both nasal cavities, characteristic of nasal polyps that do not bleed on touch, with associated mucopurulent sinonasal discharge. No other mass was seen.

**Figure 1:**
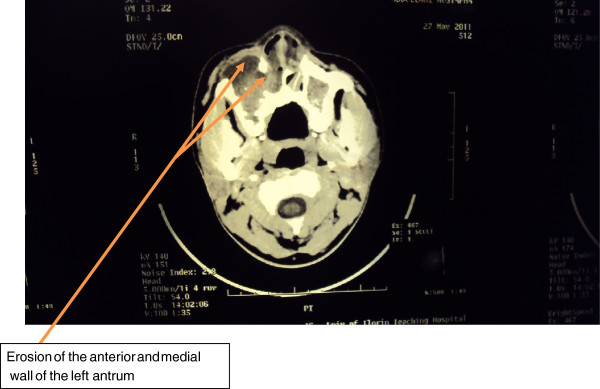
Computed tomography scan (axial) of our patient, showing the isodense lesion within the right maxillary antrum and slight erosion of the anterior and medial walls.

A CT scan of the paranasal sinuses showed an isodense lesion in both nasal cavities, maxillary antral and ethmoid, with erosion of the medial and anterior walls of the right maxillary antrum (Figure 1).

Our patient had a bilateral nasal polypectomy and intra-nasal inferior meatal antrostomy performed and blackish debris was seen in both the nasal and antral cavities. The antrostomy was widened with a Watson-Williams rasp. The operative findings were a pale glistening mass and black-greyish materials in both nasal and antral cavities, and mucopurulent aspirate from the right antrum.

The surgical specimens were sent for fungal studies and histopathology. Fungal studies yielded trichophyton mentagrophytes and the histopathology result showed inflammatory polyps. Our patient was treated with terbinafine 250mg daily for two weeks, within which time the symptoms subsided. Our patient failed to turn up for follow-up. Our patient presented again four months after surgery with a recurrent right cheek swelling; an X-ray of the paranasal sinuses showed opacification of both maxillary sinuses with erosion of the anterior and medial walls as earlier noted.

Our patient had a bilateral antral washout with warm saline under local anesthesia using the previously made antrostomy. Our patient was then given a trial of flumetasone and clioquinol (Locacorten®-Vioform®; double dilution) intra-antral application after informed consent was obtained. A nasal spray with 4% plain xylocaine was applied to the nasal cavity; flumetasone and clioquinol solution was then applied with our patient in a sitting position and neck slightly extended. An antral cannula was introduced into the maxillary antrum through an old inferior meatal antrostomy (surgically created fistula). Aspiration of the antral content was performed with subsequent introduction of the agent (flumetasone and clioquinol) with a volume of about 5mL into the cavity. An initial pre-application photograph was also obtained from our patient after informed consent was granted. This was repeated serially weekly for six weeks and close follow-up and monitoring of our patient were performed. After the first dose, there was remission of the right cheek swelling, but persistent nasal discharge. After the second dose the nasal discharge stopped with continuous regression of the mass.

A third dose was administered, and our patient successfully presented for the fourth, fifth and sixth dose. An antral aspirate yielded 3.6mL of straw-colored fluid that was sent for cytology and fungal studies. The results of these studies were negative for fungal and bacterial growth. There was improvement in our patient’s symptoms such as nasal clearance and facial pain, which reduced significantly. Our patient has been symptom free for the past four months after administration of the medication intra-antrally.

## Discussion

CRS has a significant impact on the quality of life and health burden in the adult population [4]. The impact of the disease on quality of life, as measured by Short-Form 36 (SF-36) questionnaire scores, is comparable to or worse than that of other chronic conditions such as chronic obstructive pulmonary disease, congestive heart failure and back pain [5]. Systemic anti-fungals have significant side effects, particularly with regard to hepatic and renal toxicity; topical fluconazole is a member of the new class of triazole anti-fungal agents and is a potent and specific inhibitor of fungal sterol synthesis. Its activity has been demonstrated against opportunistic mycosis such as infection with *Candida* spp. including intra-cranial infections with *Microsporium* spp., and with *Trichophyton* spp. It can also be used for systemic infections including systemic candidiasis, coccidioidomycosis and cryptococcosis. It is highly specific for fungal cytochrome p-450 dependent enzyme. Its major route of excretion is renal, with about 80% appearing in urine unchanged. To the best of our knowledge, its use in paranasal sinus fungal infection has not been previously established in the literature. Previously, other researchers have documented the use of amphotericin B. Topical amphotericin is expensive and also associated with potential adverse events [6]. With the potential for fungus to be a common mediator of CRS, and a patient population of over 60 million in the United States of America and European Union, it is essential that the adverse effects of anti-fungals for CRS are well documented prior to the widespread introduction of this form of therapy [7].

Previously, other authors proposed an argument against the use of topical anti-fungals based on the fact that fungi are ubiquitous in the sinonasal cavities, including in normal subjects. However, it has been argued that there may be a specific immune response driven by fungal antigens in patients with CRS [7]. In light of these conflicting results, it is difficult to draw any definitive conclusions for the use of topical anti-fungals at present. Previous studies have tried once-weekly treatment with fluconazole in onychomycosis [8] and tinea capitis [9].

Disease severity was measured with four widely accepted score systems. First, a modified CT score developed by Lund and Mackay [10]; the computed tomography that our patient had prior to the surgery was in support of the clinical diagnosis. Second, a modified rhinosinusitis symptom score by Lund and Kennedy [11] using the symptoms score that our patient had before and after treatments has shown a significant improvement after the start of treatment with the topical anti-fungal agent, such as reduction in facial pain and nasal blockage. Third, a rhinosinusitis quality of life score modified after Juniper *et al*. [12], and finally, an endoscopy score for nasal polyps developed by Malm [13], which has also significantly improved ethical problems as well as the financial problems that arose with the control CT at the end of the treatment (our patient could not afford the cost of the scan). The pre-operative CT scan requested took more than a month before our patient could do the scan.

Fungal infection of the paranasal sinuses is an increasingly recognized entity both in normal and immunocompromised individuals; the retroviral screening in our patient’s case was negative and our patient was not known to have diabetes mellitus. Paranasal sinus mycosis from previous studies appears to manifest as two distinct entities, a benign or non-invasive infection and the more serious invasive infection that occurs in immunocompromised individuals; the latter is characterized by its rapid onset, ability to invade tissue and destruction of tissue (in the CT findings from our patient there was also invasion of the anterior wall of the right maxillary sinus [10]).

With the use of systemic anti-fungal (terbinafine 250mg daily for two weeks) drugs, there was recurrence of the right facial swelling after four months as against application of flumetasone and clioquinol (double dilution) weekly for six weeks. There was no recurrent facial swelling after six months of follow-up.

## Conclusions

In summary, use of topical anti-fungal agents for fungal sinusitis is better for patients who have had clearance or debulking of fungal debris in the sinuses, and will help minimize the toxicity associated with systemic anti-fungal agents.

## Consent

Written informed consent was obtained from the patient for publication of this manuscript and any accompanying images. A copy of the written consent is available for review by the Editor-in-Chief of this journal.

## Competing interests

The authors declare that they have no competing interests; this research was funded wholly by the authors.

## Authors’ contributions

ADD: conceived the idea of writing this report after operating on our patient, and also revised it critically for important intellectual content. OAA: involved in the management of our patient, design and drafting of the manuscript. BSA: involved in drafting the manuscript and revising the manuscript for intellectual content. SS: involved in revising the manuscript for intellectual content. OAK: involved in management of our patient, creating the figures and drafting the manuscript. All authors read and approved the final manuscript.
